# Abdominal impacts of handlebar injuries in the PIPER child model: a prevention study

**DOI:** 10.3389/fpubh.2024.1429274

**Published:** 2024-09-13

**Authors:** Christoph Arneitz, Nico Erlinger, Corina Klug, Simone Oliver Senica, Thomas Kuenzer, Peter Spitzer, Georg Schalamon, Johannes Schalamon

**Affiliations:** ^1^Department of Pediatric and Adolescent Surgery, Clinical Center Klagenfurt, Klagenfurt, Austria; ^2^Safe Kids Austria, Graz, Austria; ^3^VSI—Vehicle Safety Institute, Technical University of Graz, Graz, Austria; ^4^Institute for Medical Informatics, Statistics and Documentation, Medical University of Graz, Graz, Austria; ^5^Department of Trauma Surgery, Clinic Diakonissen Schladming – Teaching Hospital of the Paracelsus Medical University, Schladming, Austria

**Keywords:** handlebar, handlebar grip sample, handlebar injury, pediatric, grip protection, child safety

## Abstract

**Aim:**

Analysis of data from bicycle accidents reveals that handlebar impacts are a significant cause of injury, particularly among children. Despite existing safety regulations, such as helmet requirements, little attention is given to abdominal injuries. The aim of this study is to investigate the influence of handlebar ends on abdominal loading during bicycle crashes.

**Methods:**

This study delves into the impact of five different handlebar designs on abdominal injuries during bicycle crashes, using finite element simulations with detailed Human Body Models (HBMs) of a six-year-old child (PIPER child model, Version 0.99.0). Four impact locations were identified in the injury scenario, selected according to the anatomical location of the most commonly injured organs, liver, pancreas, spleen and abdomen.

**Results:**

Grip design features, such as shape and rigidity, significantly influence injury outcomes. Grips designed specifically for children demonstrate superior performance in reducing abdominal loading and injury metrics compared to standard grips. The highest injury potential was seen in a damaged handlebar end.

**Conclusion:**

These findings underscore the importance of improved handlebar designs and standardized safety measures, especially for children. Implementation of such measures could mitigate the significant health and economic burden associated with handlebar-related injuries and enhance overall bicycle safety for children.

## Highlights

- Handlebar impacts are a significant cause of injury, particularly among children.
- Grips designed specifically for children demonstrate superior protection.
- Improved handlebar design reduces abdominal loading and injury metrics.
- Implementation of standardized safety measures against abdominal injuries required.

## 1 Introduction

In 2021, a total of 13.5 million bicycles were produced in the EU, indicating an increase of 11% compared to 2020 (1). Demand was even higher than supply. Many retailers were already sold out and there has been little change in demand worldwide in 2023. The bicycle is apparently experiencing a new all-time high, and bicycle accidents are also expected to rise.

Bicycle injuries are described as the second most common injury associated with consumer products in children (2). Although handlebar-related injuries constitute only a small percentage of all bicycle accidents, they can have life-threatening consequences. The force transmitted through a small cross-sectional area, such as the end handlebar ends, can cause significant injuries, even at low speeds (3). According to a recently published systematic review, including 138 articles with 1,072 children the most commonly injured solid organs are the liver (25.5%), pancreas (18.7%) and spleen (9.6%); the abdominal wall was affected in 14.5% (4). Injuries caused by the bicycle handlebar are often underestimated and require careful clinical evaluation (5). According to the 2021 Research Report of the Styrian Injury Surveillance System (STISS), Austria is affected by about 8,000 bike accidents per year in children and adolescents (0–18 years old) and about 600 are injured by handlebars (6). The majority of cases involve boys (74%), and the average age in our patient group is 9 years; the core age group consists of 5 to 14 years of age, with a proportion of about 83 percent. The handlebars are the main impact area on bicycles. Children are injured with handlebars or brake levers in the thoracic, abdominal or pelvic region in 66% of bicycle and scooter accidents according to the STISS accident data analysis (6). Of these injured children, 19% needed hospitalization and 12% suffered serious injuries, such as internal organ damage, fractures or severe bruises. The most frequent injury mechanism was a fall directly at the end of the turn handle (52%), which has already been described in previously published reports (6, 7).

The current regulations mainly focus on the head, such as the compulsory wearing of helmets for children, while little attention is paid to abdominal injuries (8). The current standard ISO 8098 on safety requirements for bicycles for young children, which is mandatory in the European Union, contains relatively basic requirements for the handlebar end (with a minimum diameter of 40 mm and covering the handlebar tube) for bicycles used by children aged 4 to 8 (9). There are no specific requirements for handlebars in the applicable standards (ISO 4210) for bicycles for adolescents and adults (10).

Only a limited number of studies on the interaction between the ends of handlebars and the abdomen were found in our literature review. Arbogast et al. presented an optimized handlebar end, consisting of a spring-damper element (7). Although a variety of different handlebar ends and specialized impact protections are available commercially, no studies have been found focusing on a comparison, including injury measurements. The aim of this study is to investigate the influence of handlebar ends on abdominal loading during bicycle crashes using finite element (FE) simulations with detailed Human Body Models (HBMs).

## 2 Materials and methods

The circumstances of bicycle crashes related to handlebars were analyzed by the Research Report of the Styrian Injury Surveillance System (STISS) (6). To assess the injury mechanism, a questionnaire was sent out to all parents of the children who presented in two Austrian hospitals between 2015 and 2020. Over a period of six years, a total of 206 children following handlebar-related bicycle accidents were filtered out from the accident database of pediatric and adolescent surgery with an annual average of 45 accidents (6). Severe injuries were most common with mountain biking, accounting for 40% of cases.

**Figure 1:**
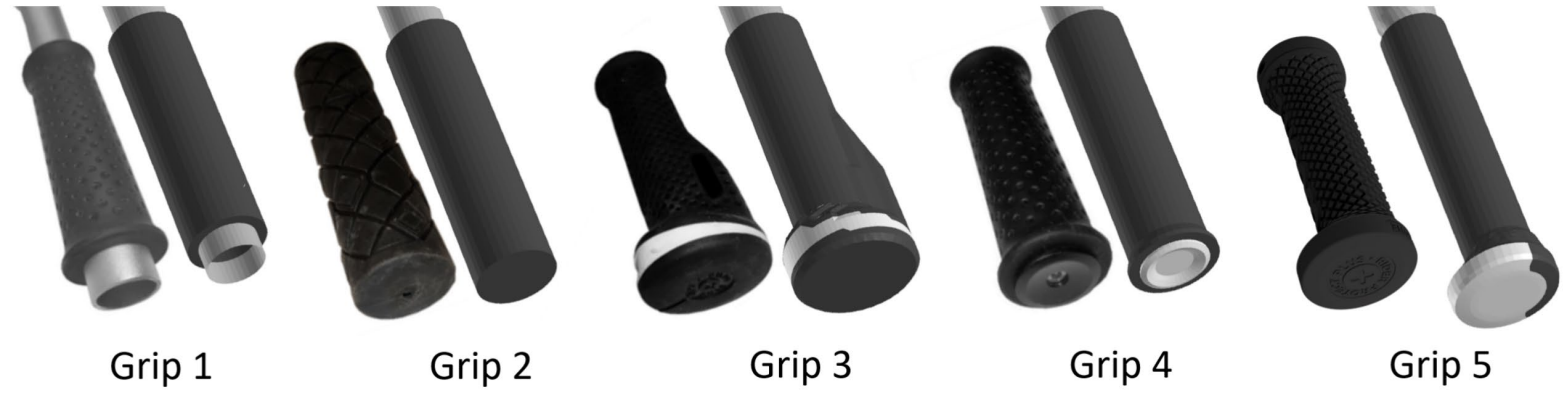
Finite element simulation setup and modelling: 5 different grips that reflect the ends of common handlebars.

The majority of the handlebar ends (72.7%) were equipped with impact protection, however, 18.2% were damaged already prior to the crash and in 9.1% the impact protection was not present. Therefore, simulations were performed with five different handlebar ends, which should reflect the most common models (Figure 1).

Grip 1 was modelled to simulate a damaged end in which the metal end of the handlebar had cut through the rubber material, resulting in an exposed tube. To assess the normal impact protection of current handlebars we chose three commercially available grips (Grip 2–4). Grip 2 has a flat end with a no specific protection. Grip 3 was designed specifically for protection with the largest diameter of all grips and is already in use. Grip 4 has a plastic cap mounted at the end of the tube, which is often used for protection. Grip 5 is a new development specifically intended for children riding mountain bikes (woom^®^, Wien, Austria). As with Grip 3, Grip 5 was designed with a handlebar end larger than previously customary in the mountain bike sector to improve protection and ergonomy (Table 1).

**Table 1:** Handlebar ends used in the study (* the diameters of grips 3 and 5 are only approximate because they are not rotationally symmetric).

	Handlebar end	Outer diameter of end [mm]
Grip 1	Damaged end, exposing metal tube of handlebar	22
Grip 2	No specific protection	30
Grip 3	Composite of rubber and plastic, asymmetrically shaped	Approx. 46*
Grip 4	Plastic cap mounted to tube end	26
Grip 5	Composite of rubber and plastic, asymmetrically shaped	Approx. 38 *

The methods for testing the impact of handlebars on abdominal injuries during bicycle crashes, using finite element simulations with detailed Human Body Models (HBMs), have been previously described in a study focusing on generic handlebar designs (11). The geometry of the handlebar and the handlebar ends (Grip 2 and Grip 4) was first created in Solidworks 2018 based on three-dimensional laser scans (FARO Quantum Max, FARO Europe GmbH, Deutschland). The geometry of all grips was then imported using the IGES format into ESI Visual-Crash 2018 In this software a mesh primarily composed of hexahedral elements with a target size of 2.5 mm was created. A first-order Ogden hyperelastic material model was assigned to the grips based on material properties fitted to data from a single grip’s datasheet (μ = −0.000327, α = −4.53). A density of 1,000 kg/m^3^ was also applied. For grips including plastic parts, also a datasheet of one grip was used in order to obtain the material parameters of the elastic–plastic material model MAT024. Both material responses were checked using a single-element setup by comparing engineering stress–strain curves. Due to the absence of datasheets or material tests for the other grips, the same material properties were employed for all grip models in the simulation. Fully integrated solid elements were applied to the grips (e.g., ELFORM 2 formulation). The handlebar itself was meshed with shell elements with a thickness of 1 mm. An elastic material model with parameters corresponding to aluminium (Young’s modulus 70 GPa, density 2,700 kg/m^3^) was assigned to the handlebar component. Connection between the handlebar and the rubber grips was done with sharing nodes. The handlebar was fixed in space, except for the grip mounting area.

The detailed FE model of a six-year-old child (PIPER child model, Version 0.99.0) was utilized for the evaluation of injury metrics. Since our research focused solely on abdominal impact, we utilized the original posture of the PIPER model. To ensure comparability between the simulations with different grips, a whole crash scenario was not included due to the complexities of movement involved. The influence of clothing, such as t-shirts or pullovers, on pressure distribution during handlebar impact was assumed to be minor, therefore it was not considered in the simulations. A previous study showed that varying the friction coefficient between the grip and the PIPER model in the range of 0.2 to 0.6 had no considerable influence on the evaluated injury metrics (11). A constant friction coefficient of 0.3 was applied, neglecting dynamic friction.

All simulations were calculated in LS-DYNA MPP R13.0.0 (ANSYS, Canonsburg, PA, USA) in single-precision. The data evaluation in the post-processing was carried out using a dynamic simulation analysis of the numerical results (Dynasaur, GitLab 2023). For calculating the injury metrics, the contact force between the grip and the trunk and the maximum principle strains (MPS) of the abdominal wall and multiple organs were used. Furthermore, beam elements (1 DOF generalized spring) with neglectable rigidity (1*10^–12^ kN/mm) were attached to each node of the PIPER child model mesh in the impacted regions to measure the indentation. The beam elements were oriented perpendicularly to the undeformed surface of the abdomen. The second nodes were coupled to a vertebra (L1 or L3) at the height of each abdominal impact in order to avoid undesirable rotational movements that cause discrepancies in the magnitude of the obtained indentation. Peak values of the respective injury metrics were obtained for each simulation.

### 2.1 Relevant crash scenarios and impact locations

The crash scenario used in our simulations was a child falling on the end of a twisted handlebar of a lateral fallen bicycle, which refers to the typical injury mechanism described previously (6, 7).

**Figure 2:**
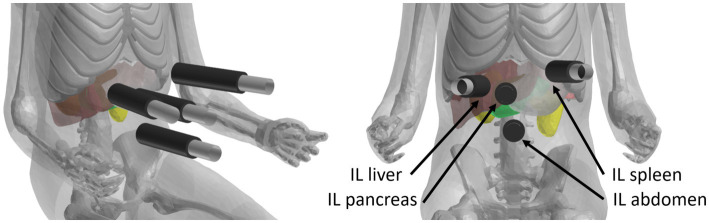
Abdominal impact locations (IL): liver (dark red), pancreas (green), spleen (light red) and kidneys (yellow) according to the most commonly injured organs (4).

Four impact locations (IL) were identified according to the most commonly injured organs published by Cheung et al. in the injury scenario and named according to these organs: IL liver, IL pancreas, IL spleen, and IL abdomen for the abdominal wall (4). The abdominal cavity bag was hidden for visibility and the following organs were highlighted in a specific color: liver (dark red), pancreas (green), spleen (light red), and kidneys (yellow) (Figure 2).

According to the low average age of 9.7 ± 3.3 years in patients with handlebar injuries we primarily focused on lower speeds (4). To investigate the influence of increasing speed we expanded our simulations to 25 km/h. Since the impact angle when falling sideways on the handlebar can vary, three different impact angles were defined.

**Figure 3:**
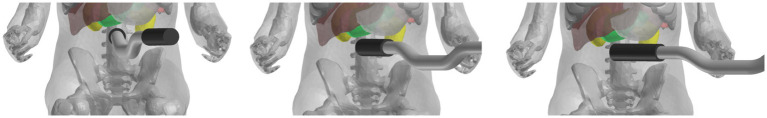
Three different angles between the abdominal wall and the outer face of the grip (left: 90°, middle: 62.5°, right: 45°).

In summary, five initial speeds (5, 10, 15, 20, 25 km/h) and three angles between the abdominal wall and the outer face of the grip (45, 62.5 and 90 degrees) were applied to each impact location (Figure 3). A total of 300 simulations were performed in the full factorial simulation matrix.

### 2.2 Simulation boundary conditions

To represent the driving speed before the crash, an initial velocity was prescribed to the PIPER child model. The movement of the handlebar was constrained in all directions, with exception of the ends of the handlebar on which the grips were mounted. So a marginal bending of the handlebar was avoided, in order to only focus on the impact of the handlebar ends and reduce possible other influences on the results. We defined a surface-to-surface contact with a friction coefficient of 0.4 (no transition to dynamic friction) between the PIPER child model and the ends of the handlebar.

### 2.3 Statistical analysis

Data were entered into an Excel 2019^®^ (Microsoft Corporation. Microsoft Excel [Internet], 2018, United States) spreadsheet and transferred to R for statistical analysis (12). Data are displayed as median and interquartile range. Multiple paired samples were compared using Friedman’s test and Wilcoxon signed-rank tests for pairwise comparisons between the handlebars with Bonferroni-Holm adjustment for multiple testing. Explorative statistical significance was defined as *p* < 0.05 (two-sided).

The comparison of different handlebar grips using a paired sample approach can provide a first assessment of the group differences, but it does not give a full picture, because it combines all the data resulting from different settings (velocity, location, angle). To investigate possible variation of the effect of the handlebar grips with respect to the other simulation parameters, we used a linear ANOVA on the logarithm of the absolute values of the outcome variables. The model used is a multiway ANOVA with saturated effects with respect to each simulation configuration, where we added terms for the handlebar grips and the interactions with the other parameters. We do not include three-way interactions with the handlebar grips both to facilitate interpretation and to compensate for the lack of replications within the factorial design. The ANOVA is summarized using proportions of sum of squares, F-statistics and corresponding *p*-values. The effects are visualized using interaction plots of the relative effects including the handlebar grips.

## 3 Results

The influence of the sensitivity properties of the handlebar ends, including shape and rigidity, on impact behavior and the resulting magnitude of the injury criteria was shown independently of the boundary conditions. The geometry, rigidity and friction coefficient between the handlebar end and the PIPER model differed. The evaluation was carried out separately for each different combination of parameters in the full factorial design, with velocities of 5, 10, 15, 20, and 25 kmph, impact locations abdomen, liver, pancreas, and spleen, and angle of 45, 62.5, and 90 degrees. Thus, for each grip there were 60 simulations for a total of 300 simulations on all handlebars (Table 2).

**Table 2:** Simulation results based on velocity, impact location and angle parameters [median (IQR)].

Characteristic	Overall	Grip 1	Grip 2	Grip 3	Grip 4	Grip 5
	*n* = 300	*n* = 60	*n* = 60	*n* = 60	*n* = 60	*n* = 60
Resultant force [kN]	2.26 (1.16–3.51)	2.19 (1.13–3.47)^b^	2.17 (1.16–3.46)^b^	2.51 (1.25–3.73)^d^	2.23 (1.16–3.42)^c^	2.19 (1.11–3.30)^a^
Max. deflection [mm]	62 (52–70)	65 (53–72)^d^	64 (52–71)^c^	60 (49–66)^a^	64 (52–71)^c^	62 (51–69)^b^
MPS liver	0.93 (0.68–1.02)	0.92 (0.67–1.01)^a^	0.92 (0.69–1.03)^a^	0.94 (0.72–1.02)^a^	0.93 (0.68–1.02)^a^	0.94 (0.68–1.03)^a^
MPS pancreas	1.51 (1.27–1.65)	1.51 (1.28–1.66)^bc^	1.51 (1.22–1.68)^b^	1.56 (1.34–1.65)^c^	1.51 (1.27–1.65)^b^	1.47 (1.21–1.63)^a^
MPS spleen	0.30 (0.20–0.48)	0.30 (0.21–0.46)^b^	0.29 (0.20–0.49)^b^	0.33 (0.21–0.54)^c^	0.30 (0.20–0.49)^b^	0.27 (0.19–0.42)^a^
MPS kidney	0.34 (0.16–0.50)	0.34 (0.17–0.50)^b^	0.33 (0.16–0.51)^b^	0.32 (0.15–0.47)^a^	0.34 (0.16–0.51)^b^	0.35 (0.17–0.47)^b^
MPS abdominal wall	1.60 (1.34–1.81)	1.72 (1.52–1.93)^d^	1.60 (1.26–1.89)^b^	1.53 (1.31–1.69)^a^	1.68 (1.44–1.84)^c^	1.52 (1.30–1.75)^a^

^abcd^ Common superscript letters denote in each row non-significant group differences in pairwise Wilcoxon signed-rank tests with Bonferroni-Holm adjustment for multiple testing (*p* > 0.05). The alphabetical ordering of the letters indicates the direction of the group contrasts, with letters in front of the alphabet indicating lower values, i.e., a < b < c < d. For all variables except for MPS Liver, the Friedman test was significant, *p* < 0.05.

**Figure 4:**
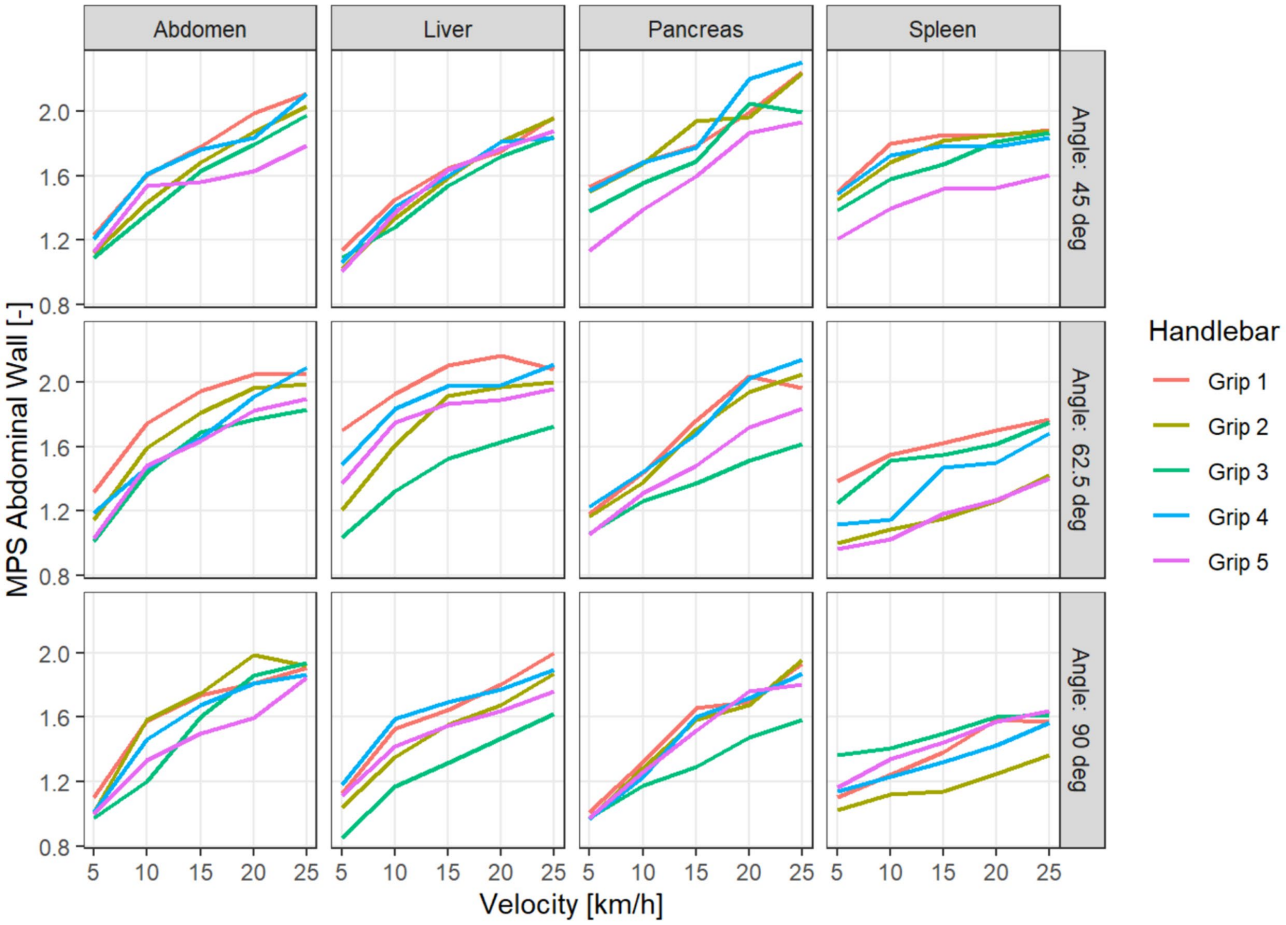
MPS Abdominal wall for different handlebar grips, depending on velocity, impact location (columns), and impact angle (in the rows).

**Figure 5:**
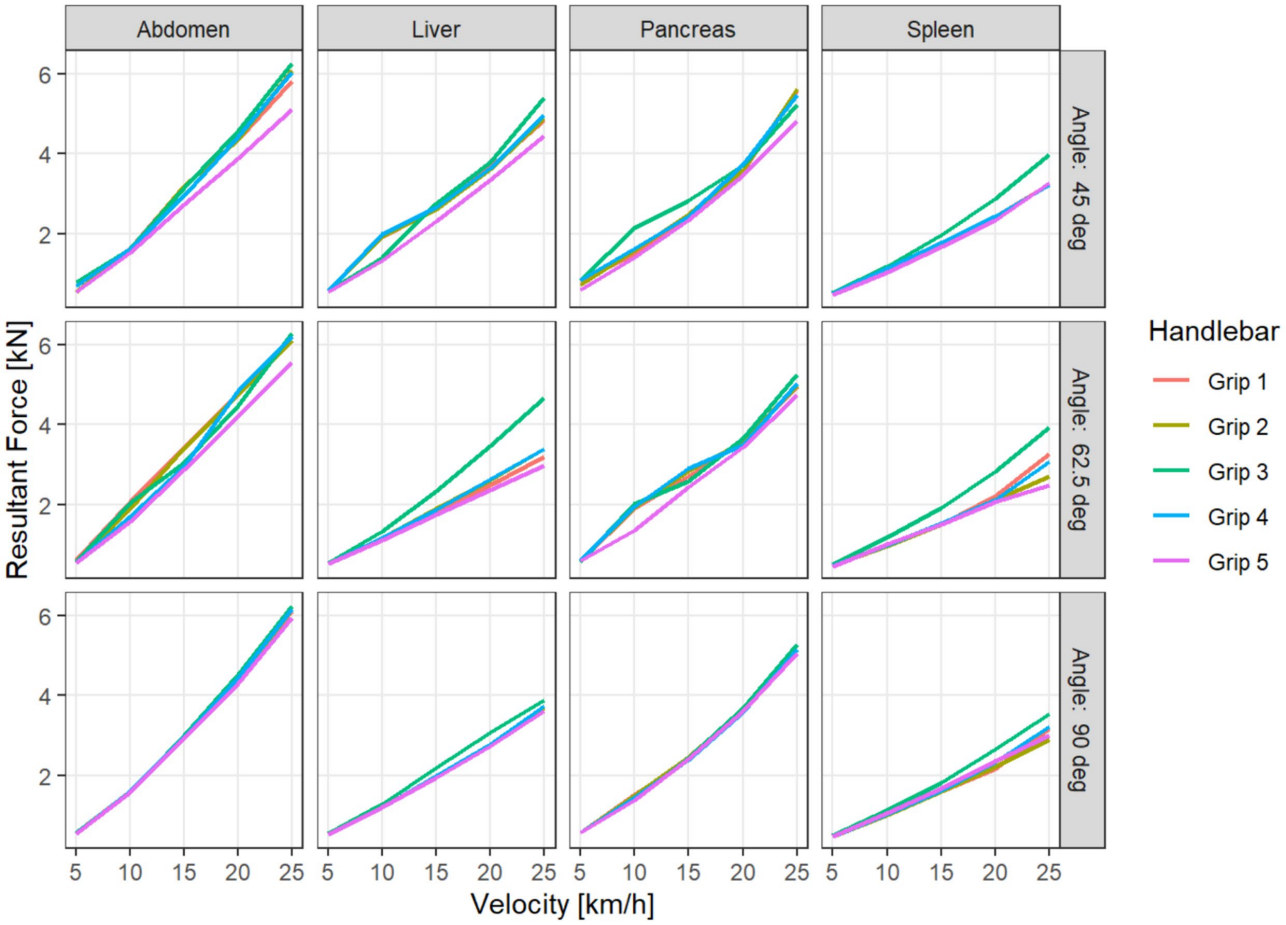
Resultant Force for different handlebar grips, depending on velocity, impact location (columns), and impact angle (in the rows).

For the impact location abdomen, all variables considered exhibited the highest absolute values. For MPS Abdominal Wall and Maximum Deflection, grip 1 generated the highest absolute values, while grip 3 produced the lowest ones (Figure 4) In the Resultant Force, grip 3 produced the highest values and grip 5 the lowest (Figure 5).

Naturally, the data exhibit the widest variability with respect to velocity, where a clear monotonous association can be seen. The relationship of the impact location and the angle to the outcome variables is more difficult to assess.

In a first analysis, we want to compare the grips 1 to 5 with different handlebar ends. Due to the full factorial design, the influence of the simulation parameters apart from the handlebar grips can be taken into account in the analysis by using a paired sample approach, where each combination of accident parameters gives rise to five paired simulations with different handlebars.

There are significant differences in variables between the handlebar grips. The Friedman test on the omnibus hypothesis is highly significant with *p* < 0.001 for all the variables considered, except for MPS Liver, where there is no significant variation with respect to the handlebar grip (*p* = 0.072). Furthermore, we used Wilcoxon signed-rank tests for pairwise comparisons between the handlebars, adjusting for multiple testing with the Bonferroni-Holm method.

The results of these pairwise comparisons vary between the variables. We note that the Resultant Force is significantly higher for grip 3 compared to any other grip, while it performs significantly better than the others for the variables Maximum Deflection and MPS Kidney. Grip 5 shows significantly lower Resultant Force, MPS Pancreas, and MPS Spleen than all other grips. For MPS Abdominal Wall, there is no significant difference between grips 3 and 5, but they both perform significantly better than the other grips. For Maximum Deflection and MPS Abdominal Wall, grip 1 performs significantly worse than all other grips (Table 2). Taking relative differences instead of absolute differences did not qualitatively change the results of the analysis.

**Figure 6:**
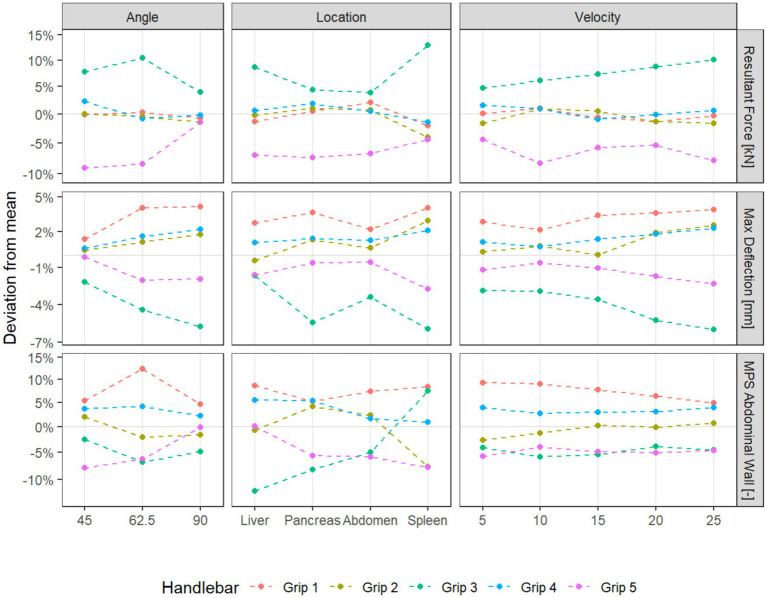
Interaction plots of handlebar grips with other simulation parameters for selected variables.

To investigate possible variation of the effect of the handlebar grips with respect to the other simulation parameters velocity and location, we used a linear ANOVA on the logarithm of the absolute values of the outcome variables. The model used is a multiway ANOVA with saturated effects with respect to each simulation configuration, where we added terms for the handlebar grips and the interactions with the other parameters. We do not include three-way interactions with the handlebar grips both to facilitate interpretation and to compensate for the lack of replications within the factorial design. The simulation parameter velocity explains the most variance for all the outcome variables, with impact location explaining the second most. The impact angle only has a weaker influence. The interactions of impact location and angle with the handlebar grips result significant, while interactions with velocity do not. Since we investigate the outcome variables on the logarithm scale, this means that the relative differences between the handlebar grips do not vary significantly with respect to velocity (Table 3). Because we are mostly interested in the effect of the handlebar grips, the interaction plots are visualized with respect to the mean outcome for given accident parameters (impact location, angle, and velocity). In these interaction plots, we can see that the interaction terms rarely cross. The interaction of handlebar grips with location shows that the variable MPS Abdominal Wall behaves differently for IL spleen than for other locations. While grip 3 performs well for the other impact locations, with a reduction of 12–19% of MPS Abdominal Wall compared to grip 1, it performs second worst after grip 1 for IL spleen, with a reduction less than 1% (Figure 6).

**Table 3:** ANOVA results: impact of handlebar grips on outcome variables across velocity, impact location and angle parameters (Df…degrees of freedom; %TSS…percentage of total sum of squares; MSS…mean sum of squares).

Variable	Term	df	%TSS	MSS	F	*p*-value
Resultant force [kN]	Velocity	4	91.0%	39.697	10,814.5	<0.001
	Location	3	6.3%	3.663	997.8	<0.001
	Angle	2	0.4%	0.325	88.6	<0.001
	Velocity × Location	12	0.7%	0.103	28.1	<0.001
	Velocity × Angle	8	0.1%	0.012	3.2	0.002
	Location × Angle	6	0.4%	0.121	33.1	<0.001
	Velocity × Location × Angle	24	0.3%	0.019	5.1	<0.001
	Handlebar	4	0.3%	0.143	38.8	<0.001
	Handlebar × Velocity	16	0.0%	0.003	0.8	0.63
	Handlebar × Location	12	0.1%	0.011	2.9	<0.001
	Handlebar × Angle	8	0.1%	0.016	4.5	<0.001
	Residuals	200	0.4%	0.004		
Max deflection [mm]	Velocity	4	72.5%	3.469	3,940.8	<0.001
	Location	3	18.4%	1.172	1,331.4	<0.001
	Angle	2	0.4%	0.034	39.0	<0.001
	Velocity × Location	12	5.8%	0.093	105.1	<0.001
	Velocity × Angle	8	0.2%	0.005	5.5	<0.001
	Location × Angle	6	0.4%	0.011	12.8	<0.001
	Velocity × Location × Angle	24	0.0%	0.000	0.4	>0.99
	Handlebar	4	1.0%	0.049	56.0	<0.001
	Handlebar × Velocity	16	0.1%	0.001	1.5	0.090
	Handlebar × Location	12	0.2%	0.003	3.4	<0.001
	Handlebar × Angle	8	0.2%	0.004	4.6	<0.001
	Residuals	200	0.9%	0.001		
MPS abdominal wall [−]	Velocity	4	62.7%	2.114	585.3	<0.001
	Location	3	2.9%	0.131	36.2	<0.001
	Angle	2	5.9%	0.397	109.9	<0.001
	Velocity × Location	12	3.7%	0.042	11.7	<0.001
	Velocity × Angle	8	0.3%	0.005	1.5	0.18
	Location × Angle	6	7.1%	0.160	44.4	<0.001
	Velocity × Location × Angle	24	1.3%	0.007	2.0	0.005
	Handlebar	4	4.9%	0.166	46.0	<0.001
	Handlebar × Velocity	16	0.2%	0.002	0.5	0.93
	Handlebar × Location	12	4.1%	0.046	12.8	<0.001
	Handlebar × Angle	8	1.3%	0.023	6.2	<0.001
	Residuals	200	5.4%	0.004		

## 4 Discussion

We analyzed the results of a simulation study of finite elements for bicycle handlebar grip injuries in children. The highest injury potential was seen in a damaged handlebar end (grip 1), while grip 3 and 5 perform significantly better than the other grips.

Grips 3 and 5 were specifically designed for children’s bicycles and were equipped with a widened and thickened handlebar end. The Resultant Force is significantly higher for grip 3 compared to any other grip, while it performs significantly better than the others for the variables Maximum Deflection and MPS Kidney. Grip 5 shows significantly lower Resultant Force, MPS Pancreas, and MPS Spleen than all other grips. In general, opposing trends were observed when comparing resultant force and deflection, based on the grip diameter. The grips with a larger diameter show a higher contact force, but distributed to a larger area of the body, leading to lower deflections of the abdomen. Furthermore, the stiffness of the handlebar ends was shown to be important previously (11). Especially, handlebar ends featuring a relatively thin and enlarged rubber lamella were shown to be prone to buckling, thus resulting in unequal distributed load on the contact area, with the bigger part of the load transmitted via the inner section supported by the handlebar tube.

The inclusion of the handlebar end 1 was motivated by the findings of the questionnaire study, which showed that in 27.1% of cases treated in the hospital the handlebar end was damaged or an impact protector was absent. Similar results were shown in a previously published study, where this kind of damage contributed to a notable proportion of lacerations (5 of 32 cases) (3).

According to these clinical findings, grip 1 showed the worst results in MPS Abdominal Wall and Maximum Deflection. Damaged handlebar ends may result in a higher likelihood of traumatic abdominal wall hernias. Traumatic abdominal wall hernias are found in 1% of patients and should be thoroughly evaluated for concomitant internal injuries (13, 14). In a recently published systematic review of traumatic abdominal wall hernia in children, the incidence of intra-abdominal injuries was 33.3% (15). Traumatic Spigelian hernia, which occurs along the Spigelian aponeurosis, is an uncommon subtype that is sporadically described in the literature (16, 17). These injury pattern reflects the high potential for injury of a damaged handlebar end and should be taken into account in prevention programs. Parents seem to be unaware of the risks and dangers of injuries caused by handlebar grips and handlebar ends. The upgrade with the “impact protection” safety element rarely or never takes place, and it must be part of the device as an equipment element from the outset. The purchase of used devices affects the younger age group. There is an urgent need to educate parents and accelerate specific training in schools.

The four different impact locations were chosen according to the most frequently injured organs in the literature (4). The present study should raise the awareness of pediatric handlebar injuries. Children are at high risk of severe injuries due to the relatively larger surface of intrabdominal organs, flexible thoracic cavity that does not cover and protect the upper abdomen, low body fat, and thin abdominal wall. Although children with intraabdominal injuries generally present with non-specific symptoms, clinicians must maintain a high suspicion level when assessing children following bicycle-related traumas. A systematic review of pediatric chest and abdominal injuries after bicycle handlebar accidents, including 138 articles with 1,072 patients, found a high incidence of misdiagnosis of thoracoabdominal injuries (4). In particular, pancreatic and intestinal injuries often manifest late and result in greater morbidity (18). In addition, there are also rare injury patterns, such as traumatic gallbladder rupture, which occurs only in 2% of all abdominal traumas reported (19).

The typical circular bruising, which can be seen in only 20.6%, was defined as a risk factor for the need for surgery (4). Even if there is no obvious organ laceration or fluid accumulation in abdominal sonography, elevated organ-specific laboratory tests can reveal intraabdominal injuries and require further diagnostic procedures, such as CT or MRI (20). In the pediatric emergency department, close observation of children after handlebar injuries and a specific institutional algorithm are highly recommended (5).

Surgical intervention is necessary for 31.5% of the patients admitted to the hospital (4). The concentrated force on a small surface area, even in slow speed bicycle crashes, was identified more than two decades ago as a critical risk for pediatric handlebar injuries (7). However, to date, there has been only one prevention study for pediatric handlebar injuries: Arbogast et al. published a new handlebar with a spring-mass-damper system that significantly reduced the forces transmitted to the abdomen of a child by half (7). The results of this study, unfortunately, have never been translated into the mass production of children’s bicycles.

Data show the greatest variability in velocity. The cycling speed significantly increases the risk of intraabdominal injuries. Briem et al. found that children’s choices for cycling speed vary depending on age and gender (21). The boy’s cycling speed increased with age, but adequate motor control was not achieved before the age of 10 (21). Therefore, children under 10 years of age are at higher risk of bicycle injuries due to serious mistakes such as stopping too late or too short stopping range. As our patients have an average age of 9 years, educational measures must focus on younger children.

The speed factor will play an even greater role in the future, as the use of e-bikes is increasing and even at a young age significantly higher speeds can be reached. A recent case report describing a 6-year-old boy who fell off an electric bike travelling at about 25 km/h revealed a penetrating abdominal injury (22). Consequently, specific restrictions and protective measures are becoming even increasingly important for the use of e-bikes.

We see approaches to prevention in both the technical (e.g., maintenance) and the behavioral area (e.g., practice and courses, personal performance level versus overconfidence). Since the assessment is based on subjective information provided by parents and not on standardized technical analyses of the individual driving devices, it is not possible to answer the extent to which special designs or specific standards can bring further improvements from available data.

The current ISO standards for handlebar ends are defined only for young children aged 4 to 8 and there are no specific requirements for bicycles of adolescents (9). The mean age of patients with intraabdominal injuries was 9.7 ± 3.3 years, while patients requiring surgical interventions were even older (10.0 ± 3.3 years). For this reason, international standards should be provided for older age groups. The injuries to the abdominal and pelvic organs caused by handlebars are a significant health hazard to children and result in considerable medical expenses. The estimated national costs associated with handlebar-related abdominal and pelvic organ injuries were $9.6 million in total hospital costs and $10.0 million in lifetime medical costs, and $503.9 million in lifetime monetized quality-adjusted life-years (23). The implementation of standards for safer handlebar designs may be a strategy to achieve health and economic advantages.

Additional modifications should be considered to reduce injuries. The shape of the brake lever should be designed in a way, especially in children’s bikes, so that even if the handlebar is rotated by 90°, it does not move into the body. In addition, a restriction on the angle of rotation of the handlebar can be considered in children’s bicycles to avoid a direct impact on the end of the handlebar. Furthermore, correct positioning of the rider on the bicycle is crucial for children’s safety and enjoyment. The optimal bicycle setup for children aged 7 to 16 was previously published taking into account their growth, flexibility, and comfort preferences (24).

### 4.1 Limitations and strengths

This study has limitations. While the full factorial design covers many accident parameters that are frequently seen in practice, it is difficult to assess how realistic these scenarios are. Because for the parameters impact location and angle only a small number of distinct values was used and the outcome variables need not be smooth in all parameters, this simulation study cannot quantify the risk of injury in an average bike accident. Further study with more granular accident scenarios is needed.

The PIPER child model’s abdominal response was validated by its developers through comparisons with physical tests: transverse belt experiments using *Post Mortem* Human Subjects (25) and bar impact tests replicating the setup used for the Q Dummy. However, the impact of the handlebar ends using in this study resulted in a smaller contact area when compared to the existing validation. Furthermore, no material tests of the handlebar ends were done. An isotropic hyperplastic material model (Ogden rubber) was fitted to material data from material specification. No strain rate dependency was included in the material model of the handlebar ends.

Strengths of this study include the use of trauma scenarios often described in a retrospective research report based on parent and patient survey (6). In addition, only one prevention study of bicycle handlebar injuries has been described in the literature (7). This is the first study applying a Human Body model for such analysis. The interdisciplinary team enabled to consider engineering and medical aspects of the analyzed problem.

## 5 Conclusion

Specially designed handlebar grips with widened and thickened ends and appropriate stiffness can significantly reduce the maximum compression of the abdominal wall and decrease the load concentration on the abdomen due to distribution to a larger area, thereby improving children’s safety. MPS Abdominal wall is reduced by up to 19% compared to damaged handlebar grips (grip 1), which have shown a high potential for injury. These results confirm the clinical findings of the last decades and must be taken into account in the international standards for the production of bicycles for children and adolescents.

## Data Availability

The raw data supporting the conclusions of this article will be made available by the authors, without undue reservation.

## References

[1] European Commission, Eurostat. Eu produced 13.5 million bicycles in 2021. Luxembourg: European Union (2022).

[2] Mehan TJ Gardner R Smith GA Mckenzie LB. Bicycle-related injuries among children and adolescents in the United States. Clin Pediatr (Phila). (2009) 48:166–73. doi: 10.1177/0009922808324952, PMID: 18936286

[3] Clarnette TD Beasley SW. Handlebar injuries in children: patterns and prevention. Aust N Z J Surg. (1997) 67:338–9. doi: 10.1111/j.1445-2197.1997.tb01986.x, PMID: 9193268

[4] Cheung R Shukla M Akers KG Farooqi A Sethuraman U. Bicycle handlebar injuries—a systematic review of pediatric chest and abdominal injuries. Am J Emerg Med. (2021) 51:13–21. doi: 10.1016/j.ajem.2021.09.043 34649007

[5] Klimek PM Lutz T Stranzinger E Zachariou Z Kessler U Berger S. Handlebar injuries in children. Pediatr Surg Int. (2013) 29:269–73. doi: 10.1007/s00383-012-3227-y 23229342

[6] Spitzer P., (2021). Verletzungen durch lenkstangen und bremsen bei unfällen mit fahrrad und scooter. In: 2021, F. ed. Forschungszentrum für Kinderunfälle im Österreichischen Komitee für Unfallverhütung im Kindesalter

[7] Arbogast KB Cohen J Otoya L Winston FK. Protecting the child's abdomen: a retractable bicycle handlebar. Accid Anal Prev. (2001) 33:753–7. doi: 10.1016/S0001-4575(00)00089-0 11579977

[8] Nadler EP Potoka DA Shultz BL Morrison KE Ford HR Gaines BA. The high morbidity associated with handlebar injuries in children. J Trauma. (2005) 58:1171–4. doi: 10.1097/01.TA.0000170107.21534.7A, PMID: 15995465

[9] International Organization for Standardization. ISO 8098:2023. Cycles Safety requirements for bicycles for young children (2023). 43 p.

[10] International Organization for Standardization. ISO 4210–2:2023. Cycles Safety requirements for bicycles (2023). 33 p.

[11] Erlinger N Schinagl M Krebs C Arneitz C Klug C. Simulation of blunt abdominal impacts caused by handlebar ends during bicycle crashes with the piper child model In: International research council on biomechanics of injury: IRCOBI Europe 2023. IRCOBI, international research council on biomechanics on injury. Cambridge, United Kingdom: Cambridge Union Society (2023).

[12] R-Core-Team. R: A language and environment for statistical computing. Vienna, Austria: R Foundation for Statistical Computing (2023).

[13] Rinaldi VE Bertozzi M Magrini E Riccioni S Di Cara G Appignani A. Traumatic abdominal wall hernia in children by handlebar injury: when to suspect, scan, and call the surgeon. Pediatr Emerg Care. (2020) 36:e534–7. doi: 10.1097/PEC.0000000000001153, PMID: 28441239

[14] Vanness G Wanner MR Chong ST Steenburg SD. Traumatic abdominal wall hernias in the pediatric population: a 13-year institutional review. Emerg Radiol. (2023) 30:51–61. doi: 10.1007/s10140-022-02101-w, PMID: 36378396

[15] Theodorou CM Stokes SC Beres AL. Traumatic abdominal wall hernia in children: a systematic review. J Surg Res. (2021) 262:181–9. doi: 10.1016/j.jss.2020.12.068, PMID: 33601272 PMC8043986

[16] Kangabam B. Traumatic spigelian hernia following blunt abdominal trauma. Cureus. (2023) 15:e35564. doi: 10.7759/cureus.35564, PMID: 37007403 PMC10063246

[17] Kropilak AD Sawaya DE. Traumatic spigelian hernia in a pediatric patient following a bicycle injury. Am Surg. (2022) 88:1933–5. doi: 10.1177/00031348221087354, PMID: 35389281

[18] Lam JP Eunson GJ Munro FD Orr JD. Delayed presentation of handlebar injuries in children. BMJ. (2001) 322:1288–9. doi: 10.1136/bmj.322.7297.1288, PMID: 11375234 PMC1120385

[19] Cacciatore CJ Pellegrin K Kashmer D Holtman NP. Handlebar injuries: not always the pancreas. Cureus. (2023) 15:e42560. doi: 10.7759/cureus.42560, PMID: 37637653 PMC10460239

[20] Wang PS Jaw TS. Easily missed pediatric handlebar injury. Pediatr Neonatol. (2023) 64:215–6. doi: 10.1016/j.pedneo.2022.03.023, PMID: 36424274

[21] Briem V Radeborg K Salo I Bengtsson H. Developmental aspects of children's behavior and safety while cycling. J Pediatr Psychol. (2004) 29:369–77. doi: 10.1093/jpepsy/jsh040, PMID: 15187175

[22] Haupt S Griffiths A. Penetrating e-bike handlebar injury. Arch Dis Child. (2023) 108:575. doi: 10.1136/archdischild-2023-325476, PMID: 37116987

[23] Winston FK Weiss HB Nance ML Vivarelli-O'neill C Strotmeyer S Lawrence BA. Estimates of the incidence and costs associated with handlebar-related injuries in children. Arch Pediatr Adolesc Med. (2002) 156:922–8. doi: 10.1001/archpedi.156.9.922, PMID: 12197801

[24] Grainger K Dodson Z Korff T. Predicting bicycle setup for children based on anthropometrics and comfort. Appl Ergon. (2017) 59:449–59. doi: 10.1016/j.apergo.2016.09.015, PMID: 27890157

[25] Kent R Lopez-Valdes F Lamp J Lau S Parent D Kerrigan J. Characterization of the pediatric chest and abdomen using three post-morten human subjects. In: Proceedings of the 22nd ESV Conference, Washington, DC. (2011).

